# Case Report: Primary Aldosteronism and Subclinical Cushing Syndrome in a 49-Year-Old Woman With Hypertension Plus Hypokalaemia

**DOI:** 10.3389/fcvm.2022.911333

**Published:** 2022-05-30

**Authors:** Lihua Hu, Wenjun Ji, Meiyu Guo, Tieci Yi, Jie Wang, Minghui Bao, Yusi Gao, Han Jin, Difei Lu, Wei Ma, Xiaoning Han, Jianping Li, Zhenfang Yuan

**Affiliations:** ^1^Department of Cardiology, Peking University First Hospital, Beijing, China; ^2^Department of Hematology, Peking University First Hospital, Beijing, China; ^3^Department of Endocrinology, Peking University First Hospital, Beijing, China

**Keywords:** hypertension, primary aldosteronism, subclinical cushing's syndrome, adrenal venous sampling, case report

## Abstract

**Background:**

Coexisting primary aldosteronism (PA) and subclinical Cushing's syndrome (SCS) caused by bilateral adrenocortical adenomas have occasionally been reported. Precise diagnosis and treatment of the disease pose a challenge to clinicians due to its atypical clinical manifestations and laboratory findings.

**Case Summary:**

A 49-year-old woman was admitted to our hospital due to fatigue, increased nocturia and refractory hypertension. The patient had a history of severe left hydronephrosis 6 months prior. Laboratory examinations showed hypokalaemia (2.58 mmol/L) and high urine potassium (71 mmol/24 h). Adrenal computed tomography (CT) showed bilateral adrenal masses. Undetectable ACTH and unsuppressed plasma cortisol levels by dexamethasone indicated ACTH-independent Cushing's syndrome. Although the upright aldosterone-to-renin ratio (ARR) was 3.06 which did not exceed 3.7, elevated plasma aldosterone concentrations (PAC) with unsuppressed PAC after the captopril test still suggested PA. Adrenal venous sampling (AVS) without adrenocorticotropic hormone further revealed hypersecretion of aldosterone from the right side and no dominant side of cortisol secretion. A laparoscopic right adrenal tumor resection was performed. The pathological diagnosis was adrenocortical adenoma. After the operation, the supine and standing PAC were normalized; while the plasma cortisol levels postoperatively were still high and plasma renin was activated. The patient's postoperative serum potassium and 24-h urine potassium returned to normal without any pharmacological treatment. In addition, the patient's blood pressure was controlled normally with irbesartan alone.

**Conclusion:**

Patients with refractory hypertension should be screened for the cause of secondary hypertension. AVS should be performed in patients in which PA is highly suspected to determine whether there is the option of surgical treatment. Moreover, patients with PA should be screened for hypercortisolism, which can contribute to a proper understanding of the AVS result.

## Introduction

Primary aldosterone (PA) is defined as the autonomous over production of aldosterone despite suppressed renin secretion. Recognizable aldosterone-producing tumors are found in about ½ the cases of PA. The remainder is produced by bilateral zona glomerulosa hyperplasia of as yet unknown etiology and very rarely, an aldosterone producing carcinoma. Clinical manifestations of PA include sodium and fluid retention and hypertension, with or without hypokalemia (1). The prevalence of PA accounts for 17 to 23% of patients with refractory hypertension (2, 3). Subclinical Cushing's syndrome (SCS) is an ACTH-independent secretion of cortisol from an adrenal adenoma that is not fully restrained by pituitary feedback, without typical clinical manifestation of Cushing's syndrome (CS) (4). SCS could cause hypertension, insulin resistance, and dyslipidemia (5). The coexisting PA and SCS are associated with increased cardiovascular morbidity and mortality (6). Previous studies have shown that the incidences of coexistent PA and SCS range between approximately 10 and 20% (6). Most of these patients had adenomas that secreted both cortisol and aldosterone simultaneously, i.e., aldosterone- and cortisol-cosecreting adrenal tumors (7–9). However, it is difficult to make the correct diagnosis in patients with concurrent PA and SCS (10). Thus, we present a case of coexistent PA and SCS to summarize the diagnosis and management of this disorder.

## Case Presentation

A 49-year-old woman of Han nationality was admitted to our hospital due to fatigue with increased nocturia for 3 years, which were worsen with decreased serum potassium for half a year. Three years ago, the patient appeared fatigued with increased nocturia and high blood pressure (BP, 170/140 mmHg). She was treated with a temporary prescription of nifedipine, captopril, spironolactone and metoprolol to control her BP. Six months ago, the patient's symptoms progressively worsened, and she was found extremely low serum potassium (2.8 mmol/L). Her serum potassium was still low even after potassium supplementation. Thus, she went to our hospital for further examination and treatment. The patient had a history of severe left hydronephrosis half a year prior and was treated with left kidney-bladder DJ tube placement. She denied using corticosteroids. Members of her family had no history of hypertension or malignant tumors.

**Figure 1:**
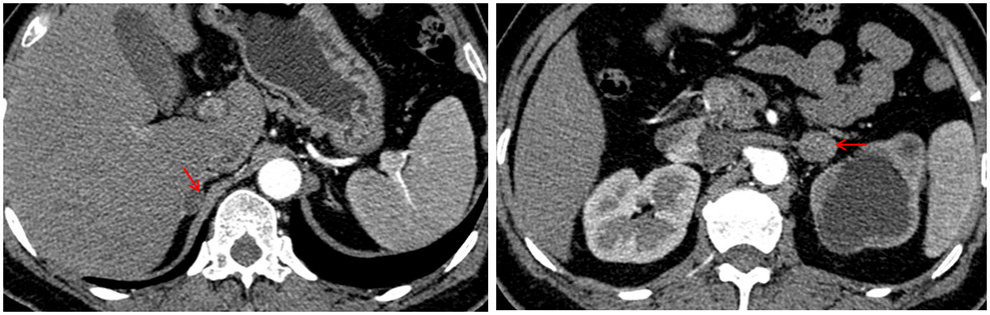
Adrenal enhanced computed tomography (CT) image showing a 16 × 13 mm right adrenal tumor and a 20 × 18 mm left adrenal tumor (red arrows).

At the time of admission, physical examinations revealed a BP of 124/82 mmHg, heart rate of 90 beats per min and respiratory rate of 20/min. Her body mass index (BMI) was 26.7 kg/m^2^. There was no physical sign of Cushing's syndrome, such as skin atrophy, buffalo hump, red striae of skin, or moon face. There were no specific findings on chest, heart or abdominal examination. The routine blood test showed that the hemoglobin concentration was 90 g/L. Biochemical tests showed low serum potassium (3.01 mmol/L). After stopping potassium supplementation, serum potassium was extremely low (2.58 mmol/L) with high urinary potassium (71 mmol/24 h). Kidney function tests showed normal serum creatinine (68.72 μmol/L) with a relatively low estimated glomerular filtration rate (89.914 ml/min/1.73 m^2^). The results of routine blood tests, liver function, blood lipids, blood glucose, thyroid function, plasma epinephrine and plasma norepinephrine were normal. No abnormalities were observed in the twelve-lead electrocardiogram, echocardiography, carotid artery ultrasound or extremity artery ultrasound. Double renal ultrasound showed severe hydronephrosis in the left, left renal atrophy (considering the stenosis of the pyeloureteral junction) and compensatory enlargement of the right kidney. Color doppler ultrasonography of renal arteries showed that there was no sign of stenosis in the trunk of both renal arteries, and there was no blood flow filling in the left kidney. An adrenal enhanced computed tomography (CT) scan revealed one rounded, homogeneous, low-density and strengthenable mass in each adrenal. The mass in her right adrenal gland was approximately 16 × 13 mm, while the mass in her left adrenal gland was 20 × 18 mm (Figure 1).

Such a young woman with hypertension plus hypokalaemia was suspected of having PA. Further screening for PA was performed (Table 1). The data showed a high plasma aldosterone concentration (PAC) and low plasma renin activity (PRA) in the supine position. The PAC remained high in the standing position; however, the aldosterone-to-renin ratio (ARR) did not exceed 3.7. She then underwent a captopril challenge test which did not show a suppressed level of plasma aldosterone concentration. The patient had hypokalaemia and left kidney atrophy, which caused false negatives for ARR. A diagnosis of PA was of no doubt.

**Table 1:** The RAAS tests and captopril challenge test.

		**Supine**	**Standing**	**Captopril test**
Normal ranges	PRA (mU/L)	2.8–39.9	4.4–46.1	_
	PAC (ng/dL)	3.0–23.6	3.0–35.3	_
	ARR	<3.7	<3.7	_
Preoperative	PRA (mU/L)	4.6	14.1	22.9
	PAC (ng/dL)	46.7	43.2	36.6
	ARR	10.15	3.06	1.60
Postoperative	PRA (mU/L)	12.2	45.2	122.2
	PAC (ng/dL)	9.41	11.2	8.74
	ARR	0.77	0.25	0.07

*PRA, plasma renin activity; PAC, plasma aldosterone concentration; ARR, aldosterone-to-renin ratio*.

*A positive diagnostic criterion for captopril test was aldosterone suppression was <30%*.

The results of cortisol rhythm and dexamethasone suppression tests are shown in Table 2. the circadian rhythm of cortisol was blunted and the adrenocorticotropic hormone (ACTH) circadian was normal. The baseline of 24-h urinary free cortisol (UFC) was 524.03 μg (normal range: 370–539 μg/24 h). An overnight dexamethasone suppression test (ODMST) showed that the plasma total cortisol (PTC) level-8 am was > 1.8 μg/dL, suggesting PTC level was not suppressed. Low (2.25 mg) and large dose (8 mg) dexamethasone suppression tests (DMST) further revealed that the PTC level-8 am was > 1.8 μg/dL and UFC was > 20 μg/24 h, showing neither serum nor urine cortisol level was suppressed. She was diagnosed with ACTH-independent secretion of cortisol. Given the absence of Cushing's classical signs, She was diagnosed as having SCS.

**Table 2:** Cortisol circadian and dexamethasone suppression tests.

		**PTC (μg/dL)**			**ACTH (pg/ml)**			**UFC (μg/24 h)**
		**8 am**	**4 pm**	**0 am**	**8 am**	**4 pm**	**0 am**	
Normal ranges		4.4–19.9			7.2–63.3			370–539
Preoperative	Baseline	15.76	10.74	7.47	17.07	6.46	2.25	524.03
	1 mg ODMST	7.47	_	_	_	_	_	_
	2.25 mg DMST	7.57	_	_	1.34	_	_	540.75
	8 mg DMST	7.92	_	_	1.25	_	_	693.99
Postoperative	Baseline	19.95	11.18	7.88	29.35	7.72	5.49	774.96
	2.25 mg DMST	7.52	_	_	1.02	_	_	438.76

*ODMST, overnight dexamethasone suppression test; DMST, dexamethasone suppression test; PTC, plasma total cortisol; ACTH, adrenocorticotropic hormone; UFC, urinary free cortisol*.

*A positive diagnostic criterion for an overnight DMST is that the PTC level-8 am was >1.8 μg/dL*.

*A positive diagnostic criterion for small and large dose DMST is that the PTC level-8 am was >1.8 μg/dL or <50% of baseline PTC and UFC was >20 μg/24 h*.

Sequential adrenal venous sampling (AVS) without ACTH stimulation was performed. Catheters for venous sampling were placed through a median cubital vein puncture. Blood was collected from the right adrenal vein (AV), left AV, and a distal end of the inferior vena cava (IVC), respectively. The results of AVS are shown in Table 3. The concentrations of plasma cortisol, aldosterone and epinephrine were measured in specimens from both AV and IVC. The selectivity index (SI), cortisol of AV/cortisol of IVC, was > 2, suggesting successful adrenal venous catheterization. Notably, the aldosterone concentration in the right adrenal vein was markedly higher than that in the LAV. The ratio of aldosterone/epinephrine in the right adrenal vein to aldosterone/epinephrine in the left adrenal vein was 23.17, which was >2, suggesting excess secretion of aldosterone from the right adrenal mass. Additionally, the cortisol concentration in LAV was similar to that in RAV. According to Young's criteria, there was no dominant side of cortisol secretion. Thus, the findings of AVS showed that the right adrenal gland was responsible for aldosterone hypersecretion. Moreover, there was no dominant side of cortisol secretion.

**Table 3:** Results of adrenal venous sampling.

	**Cortisol (μg/dL)**	**Aldosterone (ng/dL)**	**Epinephrine (pmol/L)**	**Aldosterone/Cortisol**	**Aldosterone/Epinephrine**	**Selectivity index**
IVC	6.71	34.10	21.10	5.08	1.62	
LAV	101.60	190.50	3,079.50	1.88	0.06	15.14
RAV	87.89	6,500.30	4,664.20	73.96	1.39	13.10
RAV: LAV ratio (right/left)	0.86	34.12	1.51	39.34	23.17	

*IVC, inferior vena cava; AV, adrenal vein*.

*Selectivity index: cortisol of AV/cortisol of IVC*.

A laparoscopic right adrenal tumor resection was performed half a month later. After the operation, the patient was rechecked with the captopril test (Table 1). The postoperative PAC level decreased to 9.41 ng/dL and PRA was 12.2 mU/L. The high PRA and mild hypertension might be related to left kidney atrophy. Her BP was 125/80 mmHg controlled by 75 mg irbesartan every day. The operation was successful. Her serum potassium returned to normal without any supplementary treatment, and 24-h urinary potassium levels were significantly lower than that at admission, which suggested that the operation was successful. Postoperative cortisol rhythm and levels did not return to normal due to the non-dominant cortisol secreting side (Table 2). Given the patient's absence of Cushing's classical signs, she did not receive medication. She was advised to follow up in outpatient clinics after discharge.

Pathological examination revealed that the right adrenal tumor was a 2 cm × 1.7 cm × 1 cm and bright yellow adenoma, consisting of both clear and compact cells, with the former cell type being dominant. Immunohistochemistry of the right adrenal mass revealed positive immunostaining for inhibin-α, Syn and negative immunostaining for CgA, which are the immunophenotypes of classic adrenocortical adenomas (11).

Two months after discharge, we followed up with the patient by telephone. The patient did not complain of any discomfort. Her BP remained in the normal range while taking irbesartan alone and serum potassium remained normal without any medication. Moreover, her preoperative and postoperative blood glucose and lipid were also normal.

## Discussion

Here, we present a case of a 49-years-old woman with hypertension plus hypokalaemia who had bilateral adrenal tumors, in which the right one secreted aldosterone and both secreted cortisol. The diagnosis was supported by endocrine findings and AVS. Finally, the right adrenal tumor was diagnosed as adrenocortical adenomas by immunohistochemical analysis. The patient lacked typical Cushingoid features and had many factors that influenced ARR, which posed a challenge in clinical practice to establish an accurate diagnosis. Furthermore, the interpretation of AVS findings in the patients with PA plus SCS is also challenging.

Epidemiological studies have shown that PA is the most common cause of secondary hypertension (12). The prevalence of PA in all hypertensive patients was 5%−13%, and its prevalence gradually increased with higher BP (1, 13). It is estimated that there are at least 15 million patients with PA in China. However, most patients have not been diagnosed in a timely and accurate manner. In addition to causing hypertension, patients with PA have a high risk of atrial fibrillation, metabolic syndrome, and cardiovascular and cerebrovascular events (14, 15). Studies have shown that PA patients treated by removal of the offending adenoma have better cardiovascular and renal prognoses than PA patients treated medically. Compared to patients with essential hypertension, PA patients with the same severity and duration of hypertension tend to have worse cardiovascular and renal pathology (16). Therefore, it is important for the early diagnosis and treatment of PA. Guidelines have recommended that patients with hypertension <40 years old, refractory hypertension, hypokalaemia or spontaneous hypokalaemia caused by diuretics, hypertension with adrenal incidental tumor, or sleep apnea syndrome are required for PA screening (17). However, only a small number of PA patients had hypokalaemia, and approximately 50% of patients with aldosterone adenoma had hypokalaemia, while only 17% of idiopathic aldosteronism patients presented with hypokalaemia (18, 19). Thus, the sensitivity of hypokalaemia in the diagnosis of PA was still low. The guidelines recommend plasma ARR for PA screening (17). However, due to the diversity of PA symptoms and ARR affected by many factors, its diagnosis is still challenging.

In this case, the middle-aged woman had hypertension and she was required to take four antihypertensive drugs to achieve normal BP. Combined with spontaneous hypokalaemia and renal potassium loss, PA should be considered. Renin-angiotensin-aldosterone system (RAAS) tests showed a high PAC and suppressed PRA. Moreover, the captopril test was positive. However, the ARR was negative, which was not sufficient to support PA. It is wellknown that ARR is influenced by many factors (17), such as beta receptor blockers, diuretics, angiotensin-converting enzyme inhibitors, angiotensin receptor blight agents, calcium channel blockers, renal insufficiency, renal vascular hypertension and serum potassium. The guidelines recommend maintaining serum potassium above 4.0 mmol/L when using the RAAS test. If patients were highly suspected of having PA, multiple ARR tests and a PA diagnosis test (including saline infusion test, captopril test, oral high-sodium diet, fluorohydrocortisone test) were required. In our case, the patient had hypokalaemia and left renal atrophy, which would lead to a false-negative ARR. ARR might be higher if corrected. Therefore, the diagnosis of PA was established. According to the guidelines, the imaging examination and AVS were further performed for positioning diagnosis. AVS is the gold standard for identifying unilateral and bilateral adrenal lesions in patients with PA. When PA patients choose surgical treatment, AVS should be performed to identify unilateral (often aldosterone adenoma) or bilateral adrenal lesions (often idiopathic aldosteronism) to guide follow-up treatment options (17).

The patient underwent AVS surgery. First, we judged whether the blood extraction was successful. Generally, the SI, that is, the ratio of cortisol in AV to cortisol in IVC, was chosen to be evaluated (20). In this case, SI > 2 suggested successful adrenal venous catheterization. Second, the lateralization index (LI) was used to distinguish the lateralization of aldosterone production. LI > 4 indicated unilateral PA. However, this patient had SCS. The cortisol levels might be affected by stress and uneven autonomous cortisol secretion, which posed challenges to the interpretation of AVS findings in these patients. Several studies have reported how to interpret AVS results in patients with PA and Cushing syndrome. Kaiyun Ren et al. (21) reported a 30-year-old man diagnosed with bilateral adrenal adenomas, left of which produced cortisol and right of which produced aldosterone, as determined by AVS and confirmed by immunohistochemical analysis. They measured the concentrations of plasma epinephrine in both AV and IVC to enable adjustment in the present patient. Yingxiao Zhang et al. (22) reported a PA and SCS case of bilateral adrenal tumors, in which the left adrenocortical tumor produced cortisol and the right one secreted aldosterone. The diagnosis was supported by endocrine findings, AVS and immunohistochemical evaluation of steroidogenic enzymes. They also identified the type of mutation in two adenomas. They chose cortisol of AV/cortisol of IVC as SI, and aldosterone/cortisol ratio as the criterion for the dominant side. Runa Acharya et al. (7) performed AVS surgery in eight patients with SCS, with dexamethasone at 0.5 mg Q6 h the day before the operation. Young's criteria were used to determine the adequacy of cannulation of the adrenal veins. Catheterization of an AV was considered successful if the plasma epinephrine (Epi) concentration in the AV was 100 pg/ml above the IVC (AV–IVC >100 pg/ml). Side-to-side (higher cortisol/lower cortisol) cortisol lateralization ratios (CLR) were also calculated. The data were analyzed using criteria from the study by Young et al. (23) of an AV/PV cortisol ratio of >6.5 on one side and ≤3.3 on the contralateral side, and CLR ≥ 2.3 to indicate a unilateral cortisol-secreting adenoma. A CLR of ≤2 was used to indicate bilateral cortisol hypersecretion. In this case, both SI and Young's criteria indicated that the adrenal vein intubation was successful. Based on previous studies, adrenaline was used as the denominator of aldosterone to determine the dominant side of aldosterone secretion, and Young's criteria were used to determine the dominant side of cortisol secretion. The AVS results showed hypersecretion of aldosterone from the right adrenal tumor with no dominant side of cortisol secretion. The immunohistochemical analysis further confirmed AVS results. Her postoperative serum potassium remained normal without any medication. However, because the patient had left kidney atrophy, PRA activation remained after the operation, resulting in high BP.

SCS is generally found when a patient with an unidentified adrenal mass underwent DMST, and it is the most common endocrine syndrome associated with an adrenal adenoma (22, 24). Many cases of PA with SCS have been reported as aldosterone/cortisol cosecreting adenomas (6). Both overt and subclinical Cushing's syndrome develop hypertension, dyslipidemia, insulin resistance and T_2_DM. Those with subclinical disease may not be tested, thus treated, for these because they are not as easily recognized as the more overt symptoms of Cushing's. Thus, such patients still closely monitor their blood glucose and blood lipid levels after hospital discharge. In this case, the results of cortisol rhythm and DMST showed ACTH-independent secretion of cortisol. Because the patient did not have typical Cushingoid features, SCS diagnosis was established. AVS further showed no dominant side of cortisol secretion. After the operation, the circadian rhythm of cortisol disappeared and her serum and urine cortisol levels were not suppressed by a low-dose DMST. Therefore, the patient was still diagnosed with SCS. Given the patient's absence of Cushing's classical signs, she currently did not receive medication. She did not receive hormonal therapy and was advised to follow up in outpatient clinics after discharge. The patient's blood glucose and blood lipids without any medication remained normal during postoperative follow-up. Long-term follow-up is still required for this patient.

There are some limitations to our study. Immunohistochemical staining for CYP11B1 and CYP11B2 in the right adrenal tumor was not performed. the immunohistochemical analysis of DHEA-sulfotransferase in the adherent tissue of the right adrenal adenoma was not performed. And, several mutations in addition to *KCNJ5* mutations related to aldosterone-producing adenoma were not detected. Thus, the diagnosis of right aldosteronoma could not be confirmed. In addition, there was no pathological explanation of the cause of excess cortisol in this case.

## Conclusion

This case gave us some inspiration. First, we should pay attention to the screening of the causes of secondary hypertension. Second, AVS should be performed in patients in which PA is highly suspected to determine whether there is the option of surgical treatment. Third, patients with PA should be screened for hypercortisolism. It can contribute to a proper understanding of the AVS result. Fourth, the interpretation of AVS findings in the patients with PA plus SCS is different from others.

## Data Availability Statement

The raw data supporting the conclusions of this article will be made available by the authors, without undue reservation.

## Author Contributions

MG, WJ, TY, JW, DL, WM, JL, ZY, and XH contributed in this patient care, diagnosis, and treatment. LH, MB, YG, and HJ collected the data. LH drafted this manuscript. DL, XH, JL, and ZY revised the final version of the manuscript. All authors have read and agreed to the published version of the manuscript.

## Conflict of Interest

The authors declare that the research was conducted in the absence of any commercial or financial relationships that could be construed as a potential conflict of interest.

## Publisher's Note

All claims expressed in this article are solely those of the authors and do not necessarily represent those of their affiliated organizations, or those of the publisher, the editors and the reviewers. Any product that may be evaluated in this article, or claim that may be made by its manufacturer, is not guaranteed or endorsed by the publisher.
