# Corrigendum: Case report: Primary aldosteronism and subclinical cushing syndrome in a 49-year-old woman with hypertension plus hypokalaemia

**DOI:** 10.3389/fcvm.2022.1055119

**Published:** 2022-11-03

**Authors:** Lihua Hu, Wenjun Ji, Meiyu Guo, Tieci Yi, Jie Wang, Minghui Bao, Yusi Gao, Han Jin, Difei Lu, Wei Ma, Xiaoning Han, Jianping Li, Zhenfang Yuan

**Affiliations:** ^1^Department of Cardiology, Peking University First Hospital, Beijing, China; ^2^Department of Hematology, Peking University First Hospital, Beijing, China; ^3^Department of Endocrinology, Peking University First Hospital, Beijing, China

**Keywords:** hypertension, primary aldosteronism, subclinical cushing's syndrome, adrenal venous sampling, case report

In the published article, there was an error in the author list, and authors “Meiyu Guo, Zhenfang Yuan” were erroneously excluded. The corrected author list, affiliations, correspondence, and citations appear below.

Lihua Hu^1^^†^, Wenjun Ji^1^^†^, Meiyu Guo^2^, Tieci Yi^1^, Jie Wang^1^, Minghui Bao^1^, Yusi Gao^1^, Han Jin^1^, Difei Lu^3*^, Wei Ma^1^, Xiaoning Han^1*^, Jianping Li^1^ and Zhenfang Yuan^3^

^1^ Department of Cardiology, Peking University First Hospital, Beijing, China

^2^ Department of Hematology, Peking University First Hospital, Beijing, China

^3^ Department of Endocrinology, Peking University First Hospital, Beijing, China

^*^Correspondence:

Difei Lu

ludifei3142@163.com

Xiaoning Han

dr_hanxn@126.com

In the published article, there was an error in the **Author contributions** section, and authors “Meiyu Guo, Zhenfang Yuan” were erroneously excluded. The corrected author contributions section appears below.

## Author contributions

MG, WJ, TY, JW, DL, WM, JL, ZY, and XH contributed in this patient care, diagnosis, and treatment. LH, MB, YG, and HJ collected the data. LH drafted this manuscript. DL, XH, JL, and ZY revised the final version of the manuscript. All authors have read and agreed to the published version of the manuscript.

In the published article, a **Funding** statement was erroneously included. The **Funding** statement has been removed from the published article.

In the published article, an **Ethics statement** was erroneously included. The **Ethics statement** has been removed from the published article.

In the published article, authors “Meiyu Guo, Zhenfang Yuan” were erroneously excluded from the Copyright section. The corrected Copyright section appears below.

“Copyright © 2022 Hu, Ji, Guo, Yi, Wang, Bao, Gao, Jin, Lu, Ma, Han, Li and Yuan. This is an open-access article distributed under the terms of the Creative Commons Attribution License (CC BY). The use, distribution or reproduction in other forums is permitted, provided the original author(s) and the copyright owner(s) are credited and that the original publication in this journal is cited, in accordance with accepted academic practice. No use, distribution or reproduction is permitted which does not comply with these terms.”

The authors apologize for these errors and state that this does not change the scientific conclusions of the article in any way. The original article has been updated.

## Publisher's note

All claims expressed in this article are solely those of the authors and do not necessarily represent those of their affiliated organizations, or those of the publisher, the editors and the reviewers. Any product that may be evaluated in this article, or claim that may be made by its manufacturer, is not guaranteed or endorsed by the publisher.

